# «Doctors must live»: a care ethics inquiry into physicians’ late modern suffering

**DOI:** 10.1007/s11019-025-10258-7

**Published:** 2025-02-05

**Authors:** Caroline Engen

**Affiliations:** 1 Neuro-SysMed, Department of Neurology, Haukeland University Hospital, Bergen, Norway; 2 Centre for the Study of the Sciences and the Humanities, University of Bergen, Bergen, Norway; 3 Division of Psychiatry, Haukeland University Hospital, Bergen, Norway

**Keywords:** Ethics of care, Late modernity, Medicalization

## Abstract

In 2023, thousands of young Norwegian physicians joined an online movement called *#legermåleve* (#doctorsmustlive) and shared stories of their own mental and somatic health issues, which they considered to be caused by unacceptable working conditions. This paper discusses this case as an extreme example of physicians’ and healthcare workers’ suffering in late modern societies, using Vosman and Niemeijer’s approach of rethinking care imaginaries by a structured process of *thinking along*,* counter-thinking and rethinking*, bringing to bear *suffering* as a heuristic device. *Thinking along*, taking the physicians’ stories and arguments literally, reveals an image of an unbearable workload. *Counter-thinking* resituates their suffering within the broader conditions of late modernity, suggesting that the root cause may lie not in the quantity of the workload itself but in its qualities and in its perceived threat to their integrity as caregivers through epistemic and moral injury and an inability to respond to this threat. In *rethinking*, the ambiguity of suffering– its dual potential as both a constraint and an opening– becomes central. Following the physicians’ own interpretations and the solutions emerging from this framing, both their suffering and that of their patients could paradoxically be exacerbated by further decentering physicians and reinforcing utilitarian, data-driven approaches. However, staying with their suffering and reinterpreting its causes opens possibilities to leverage critiques of medicalization at large and of their own suffering in particular, challenging the assumption that the weight of care must always grow heavier. From this reframing, I argue, it is possible to reclaim and reimagine care and the clinical space as a nexus of epistemic and moral privilege, recentering response-ability both relationally and socially.

## Introduction

June 2023, upon the suicide of a young hospital doctor, Norwegian physicians and healthcare workers increasingly shared on social and news media their accounts of unacceptable working conditions and their own mental health issues such as burn-out, depression, and suicidal thoughts. An online movement emerged under the name *#legermåleve* (#doctorsmustlive, “doctors must live”) (Espegren 2023; Tveito 2023), with its home in a private Facebook group intended for physicians and medical students. As of January 2024, the group counted more than 6,200 members. Although one cannot verify if all members indeed are physicians or medical students, the size of the group was substantial for Norway which is a country with a total of no more than 40,000 physicians.

When viewed from a distance, the Norwegian #doctorsmustlive movement may be hard to comprehend. At a time of war and political and economic crises in Europe and around the world, Norway was a politically stable country in a state of peace, with publicly funded universal healthcare and a highly developed and continuously growing welfare state. Norway has been among the richest countries in the world for decades, and labour rights are among the most advanced in the world, thanks to Norway’s celebrated Work Environment Act (Often 1991; Torp and Reiersen 2022). And yet, physicians all over the country began to share their stories of an unbearable work life and contemplations of suicide.

This apparent paradox calls for deeper inquiry. How can such distress emerge in a context often celebrated for its quality of life and progressive labour rights, and among a group of professionals who are traditionally regarded– and often regard themselves– as privileged? To address this question, this paper proposes an understanding of the #doctorsmustlive movement through a care ethical framework. In doing so, it seeks to place the phenomenon in a larger context of late modern healthcare, exploring its relational and structural dimensions and responding to its far-reaching implications for care practices and care practitioners. The purpose of this paper, as such, is not just to make an apparently strange phenomenon understandable but also to use it as an (arguably extreme) example of a possibly more general trend of development of healthcare in early 21st century welfare states. The fact that depression, burnout and suicidal thoughts are prevalent among physicians and nurses is not new (Chen and Meier 2021; Crudden et al. 2023; Kalmoe et al. 2019; Mata et al. 2015; West et al. 2018). Recently, the pressures and strains on healthcare professionals have gained increasing attention, with growing concerns about a mental health crisis among those working in the field. This crisis not only poses significant risks to patient care and safety but also threatens the effective functioning of healthcare institutions, as many healthcare practitioners are compelled to leave their positions due to unsustainable working conditions. I shall argue that this broader trend is related to the restructuring and reconfiguration of clinical spaces under conditions referred to as late modernity, a phase described and analyzed as one in which the dynamics and characteristics of modern institutions are both radicalized and expanded (Giddens 1990). This restructuring is characterized *inter alia* by increasing specialization, differentiation, organisation, and regulation of tasks, expertise, and institutions (Beck 1992; Giddens 1990; Hood 1991), alongside a rapid pace of technological advancement and social change (Rosa 2013). These dynamics create a landscape where change outpaces adaptation, giving rise to uncertainty and risk (Beck 1992), reflected in fragmented, transient, and continuously shifting subjectivities (Bauman 2000).

In their seminal article, Vosman and Niemeijer (2017) argued that these late modern conditions destabilize traditional roles, relationships, and responsibilities within care practices themselves. Introducing precariousness– persistent insecurity about one’s position and relationships within these systems and with each other– as a feature affecting both providers and patients alike, they asked whether the specific features and developments of late modern societies necessitate a reconceptualization of care ethics (Vosman and Niemeijer 2017). In their article they argued for a need to engage critically with these systemic pressures, advocating for a care ethics that is responsive to the dynamic and precarious nature of late modern institutions. To illustrate this, they developed and applied such a care ethical framework: a heuristic-based methodology that combines phenomenological inquiry, sociological reflection, and care ethics to analyze the lived realities of care. Through three iterative steps– *thinking along*, *counter-thinking*, and *rethinking*– and using the heuristic of precariousness, Vosman and Niemeijer demonstrated how the increasing complexity of healthcare institutions, particularly hospitals, and the diverse manifestations of precariousness among healthcare workers, can result in practices that, from the patient’s perspective, may be experienced as a breakdown or absence of care. Yet, when adopting a contextual and situational understanding of the complexity of tasks assigned to those working within late modern healthcare institutions, they were able to foreground the breadth and depth of care work contemporary healthcare workers are charged with, foregrounding moral and relational efforts embedded in these practices, even under challenging systemic pressures.

They went on to caution, “Nobody can set moral standards for the hospital by simply pointing at its telos and the subsequent moral goods which are at stake” (ibid., p. 471), arguing that any contribution from the ethics of care should be grounded in the perspective of the concrete practices in question, the positions and lived experiences of those who are involved in the practices, and the relationships and enactments of power in the concrete situation. This dual orientation– proximity to the lived experiences of care practitioners alongside reflective distancing to enable broader sociological and philosophical reflection and critique– resonates with my own positionality as both a practicing physician and a scholar of medical philosophy. The framework developed by Vosman and Niemeijer offers a space for me to contribute in this dual sense, reflecting and navigating the inherent tensions between situatedness and distance in relation to the #doctorsmustlive movement.

The objective of this paper is to continue the work set out by Vosman and Niemeijer of rethinking care imaginaries in late modern societies. The paper will follow Vosman’s and Niemeijer’s suggestion of *thinking along*,* counter-thinking* and *rethinking* the suffering and despair expressed by the #doctorsmustlive movement. Whereas the physicians (and other healthcare workers) themselves predominantly have expressed the issue as one of unbearable working conditions and notably one of time pressure, *counter-thinking* their concerns in light of late modern conditions and through the heuristic of suffering I shall argue that physicians are faced with the impossibility of living up to epistemic and moral standards, as well as professional responsibilities rooted in a different societal context when their positions, relationships, and authority were vastly different. Based on this I shall proposes a reevaluation of their concerns in terms of a lost sense of professional and vocational identity, alongside diminished experiences of caring– encompassing knowing, doing good, and responding– which together result in a pervasive loss of gratification and reward that physicians derive from their work. I shall further argue that unable to *rethink* and reimagine their professional identities and practices outside current framings, the emerging solutions risk intensifying the very pressures they aim to alleviate, further entrenching systemic constraints and exacerbating their sense of failure and disempowerment. If these claims hold, a rethinking of care imaginaries may be an urgent issue of existential importance. I shall argue that it is precisely in their suffering that an opening for such *rethinking* emerges, offering a pathway to critically engage with and transform the conditions shaping both care and the caregivers themselves.

## Methodological approach: care rethought

In this paper as well as in the literature to which it intends to contribute, the concept of care operates on several levels. First, on what we might call the objective level, the phenomenon to be analyzed and discussed– the #doctorsmustlive movement– was created by healthcare professionals (in this case, physicians) working in healthcare organisations. Secondly, the phenomenon is doubly related to care practices in that the physicians warned against unacceptable working conditions that threatened the care given to patients but also threatened the conditions for personal self-care for the physicians themselves. Thirdly, the genesis of this paper is itself rooted in care. As a practicing physician myself, I care deeply about what is presented as at stake in the #doctorsmustlive movement: the patients, my colleagues, our practices, and, inevitably, myself. Finally, the objective of this paper is to engage in critical reflection on care. Care practices were at stake in the issues raised by the #doctorsmustlive movement, but, drawing on resources from the medical humanities I shall argue that the particular way that care was imagined within the movement, was inadequate to the level of posing existential risk. Accordingly, I shall engage in the philosophical venture of *rethinking care.* The care ethics tradition provides resources for thinking across these multiple levels. While placing moral emphasis on situatedness, relatedness, and the experiential (Gilligan 1982), care ethics is also engaged with “webbed” dynamics of care as relationally interconnected and contextually embedded (Held 2006). Furthermore, care ethics include resources for exploring political and structural dimensions of care and how care is shaped by and in turn reshape power relations and institutional practices (Tronto 1993). Together, these approaches allow for a rich exploration of the #doctorsmustlive movement, including the relational, epistemic, moral, and systemic dimensions of the physicians’ concerns. For this venture, I have therefore, for the above-mentioned reasons, found it not only relevant but also *right* to draw on care ethics and care theory as both theoretical and methodological resources.

Care ethics is, however, increasingly challenged by the complexity and systemic pressures of late modernity, where caregiving practices are shaped by neoliberal influences, bureaucratic demands, and relational ruptures. This paper builds on these insights by applying Vosman and Niemeijer’s heuristic-based methodology to analyze the #doctorsmustlive movement, offering a lens to explore the relational and institutional tensions that define late modern care. Questioning the reflexivity of care ethics itself in relation to its own positioning within late modern societies Vosman and Niemeijer developed an analytical framework that attempts to provide such a reflexive space. By drawing attention to shifts in the broader sociocultural context of care, influenced by features of late modernity, they foregrounded the reordering and repositioning of both care providers and recipients and a restructuring of responsibility and response-ability. They emphasized the importance of marking out relative positions in the care setting to foreground the political character of a field. They argued that this approach may reveal the moral ambiguity of positions and helps identify “where the actual good might emerge”. Hence, Vosman and Niemeijer argued that “[c]are ethics is not about giving normative prescriptions from the outside of a practice, it is rather about looking along with practitioners at what they see, looking at what is good and bad in a practice and discerning them in the ethos” (Vosman and Niemeijer 2017). In sum Vosman and Niemeijer’s framework is particularly well-suited for analyzing late modern phenomena like the #doctorsmustlive movement due to its ability to address the interplay between systemic complexity, precariousness, and relational dynamics in care settings. Their approach bridges the gap between normative ethical inquiry and the sociological realities of late modernity, offering a robust methodological toolset to critically engage with phenomena characterized by relational and institutional tensions. Vosman and Niemeijer’s work builds on key philosophical insights into late modernity, such as Anthony Giddens’ concept of “modernity over the top” (Giddens 1990) and Hartmut Rosa’s notion of accelerated social dynamics (Rosa 2013). These theories highlight the intensification of systemic demands and the destabilization of traditional roles and identities within institutional frameworks, both of which are evident in the #doctorsmustlive movement. By incorporating these late modern conditions into their care ethics methodology, Vosman and Niemeijer provide a means of analyzing how care practices are restructured under these pressures, providing a structured approach to understanding the #doctorsmustlive movement as a symptom of broader late modern challenges.

In practical terms, Vosman and Niemeijer’s proposed to identify key case-based heuristics and then explore the case through a three-step process: first “thinking along,” then “counter-thinking,” and finally “rethinking.” The initial step involves *thinking along* with the stakeholder’s views and concerns, taking their various perspectives seriously in an effort to understand them on their own premises. The second phase, *counter-thinking*, involves a critical examination of the care setting, searching for more fitting frameworks, yet while remaining loyal to the concerns of those involved. The final phase, *rethinking*, involves reflecting on and potentially reformulating understandings based on the insights gained from the previous phases, by opening up the scope to identify more concerns, more care and more to be cared for. In this way, Vosman and Niemeijer presented a methodological approach that sought to combine the moral positioning in care ethics with sociological reflection on complex late modern institutions and, at the same time, a phenomenological approach to identifying key matters of concern and care. In their terminology, such key matters can be captured by means of “heuristic devices” (rather than making ontological claims about “essences”), and in their paper, they related in large part to the vocabulary of care theory to formulate these heuristic devices. I have followed their approach, and will in what follows propose “suffering” as a key heuristic device for the *counter-thinking* and *rethinking* of the #doctorsmustlive movement.

I have chosen suffering as a heuristic for this analysis for several reasons. First, claims of suffering are central to the phenomenon under discussion– the physicians are making claims of suffering. Second, in modern and late modern projects, alleviating suffering is a central aim (Amato 1990), with medicine itself organized around the goal of reducing suffering: diagnosing it, treating it, and even predicting it. Yet, as critiqued by the physician-philosopher Cassell (1982), this medical focus often reduces suffering to physical or measurable dimensions, potentially overlooking its deeper existential, relational and systemic dimensions, bringing us to the third reason: suffering reveals a paradox within late modernity itself, exposing limitations of the very systems designed to address it. For the critical theorist Theodor Adorno, suffering is not merely subjective pain but an objective marker of the contradictions and failings within societal structures (Adorno 1973). In this sense, suffering becomes a demand for critique– a refusal to accept the *status quo* and a call to imagine alternatives, bridging individual experiences and broader social and systemic dynamics (Geuss 2005; Schick 2009). Adorno’s insistence on lending a voice to suffering as a condition of truth provides a philosophical foundation for this approach (Adorno 1973, p. 17). Lastly, drawing on the philosophy of Emmanuel Lévinas, suffering takes on a profound ethical dimension, directly connecting it to the essence of care itself. For Lévinas, suffering, despite it’s uselessness, represents “the original opening toward what is helpful, where the primordial, irreducible, and ethical, anthropological category of the medical comes to impose itself” (Levinas 1988). For Lévinas, suffering is not merely an individual experience but a profoundly relational phenomenon– comprising a call and a response that binds sufferer and witness in a shared space where recognition, empathy, and responsibility converge. To Lévinas, this opening is not a given but a possibility, one that must be actively realized through ethical engagement (White 2012). This ambiguity of suffering, present in Lévinas thought, share resemblance to a longstanding and recurring tension between two interpretations of suffering– suffering as passivity, a state of being bound, and suffering as an opening, a task to engage with. This tension is echoed in philosophical traditions, from Aristotle’s view of suffering as a means of moral growth and Plato’s emphasis on transcending earthly struggles, to Nietzsche’s framing of suffering as essential for self-overcoming and Bentham’s utilitarian focus on minimizing pain (Aaltola 2021). Following Adorno and Lévinas, one can argue that something critical, epistemic, and moral is at stake in the #doctorsmustlive movement, and that it is through engagement with suffering itself that we may gain access to what can truly be “helpful”.

The ambiguity of and tensions within suffering makes it a particularly rich, yet challenging, concept to think with. It resists clear linguistic expression, neat categorization and parametrization, as well as definitive representation (Scarry 1985; Sontag 2003). Yet, to use suffering as a heuristic requires grappling with the impossible task of articulating it. Bearing this reductive nature of language in mind one way to understand suffering is by exploring its etymological root. Derived from the Latin sufferre, “to bear, undergo, or endure,” to suffer is to carry a burden (Rodgers and Cowles 1997; VanderWeele 2019). Another way, as suggested by Cassell (1982), is to view suffering as a state arising from the perceived threat to or disintegration of one’s sense of self as a person (Cassell 1982). Suffering, understood through these complementary perspectives, is particularly well-suited as a heuristic device in this context for several reasons. First, it aligns closely with the lived experiences and narratives of the #doctorsmustlive movement. By engaging with suffering, it becomes possible to confront both the tangible burdens faced by physicians and to think alongside these stakeholders, taking their concerns seriously and understanding their perspectives from the inside out. Second, suffering is inherently relational– it arises within contexts of unmet needs, failed expectations, and broken connections. As such, suffering provides a means to explore not just the individual experiences of distress but also the relational and institutional ruptures that underlie experiences of threat to their integrity as professionals and persons. This dual conception - suffering as both a burden and a threat to integrity - makes it a powerful lens for *counter-thinking*. It enables an exploration not only of the burden itself but also of its deeper relationship to those who bear it. The ambiguity of suffering, and the tension within it - between being bound and being an opening - makes it a powerful lens through which to *rethink*. By engaging with this tension, suffering not only reveals the constraints and challenges faced by physicians but also opens up possibilities for imagining new ways of understanding and addressing the systemic and relational ruptures inherent in late modern healthcare. In sum, suffering resonates with the integration of phenomenological, relational and systemic orientations inherent in and constituent of Vosman and Niemeijer’s framework.

To transition toward positioning myself in this analysis, suffering offers a pathway not only to critique systemic structures but also to locate myself within the tensions and possibilities of care. As Vosman and Niemeijer emphasize, care ethics is not about detached normative prescriptions but about “looking along” with practitioners to discern what is good and bad in practice. My choice to engage with suffering as a heuristic is shaped by my dual positionality as both a practicing physician and a scholar of medical philosophy. This dual perspective allows me to approach suffering not just as an abstract concept but as a lived reality that I, too, navigate within the context of late modern healthcare.

As a Norwegian physician educated in a Norwegian medical school and currently practicing part-time as a psychiatrist in specialization, my demographic profile closely aligns with the movement’s key spokespersons. I share their professional concerns as care providers and their experiences of systemic and cultural challenges in late modern healthcare. This proximity allows me to *think along* with their concerns, taking their aspirations and frustrations seriously as a starting point for analysis. At the same time, my background in philosophy (hopefully) enables me to critically interrogate these experiences, situating them within broader theoretical and societal frameworks. This reflective distance, aligned with Vosman and Niemeijer’s concept of *counter-thinking*, allows me to explore the structural and cultural forces that contribute to the suffering of fellow physicians, and in part also my own, using the heuristic device of “suffering” to bridge individual and collective dimensions of care. The *rethinking* process synthesizes these perspectives, enabling the identification of new possibilities and concerns. In this context, *rethinking* is deeply shaped by my dual positionality. As a physician, my observations and experiences offer an insider’s understanding of the weight of the burden– how it threatens professional integrity and gives rise to suffering, while simultaneously being productive of hope and imagination. At the same time, my philosophical perspective brings critical insight into how these individual experiences connect to broader societal trends and institutional dynamics.

By integrating proximity and reflective distance, I aim to address the dual challenges of professional identity and institutional critique within late modern societies. This dual perspective, as such, does therefore not merely inform the analysis but becomes integral to the methodological approach itself. By positioning myself within the reflexive framework that Vosman and Niemeijer envisioned, I will attempt to navigate the tensions of being both within and outside the care setting allowing for a grounded yet expansive contribution to the *rethinking* of care ethics in late modern contexts.

## #Doctorsmustlive

As mentioned in the introduction, in 2023, a Norwegian female junior doctor tragically committed suicide. Her death sparked a public uproar among Norwegian physicians. The physician’s widower published an appeal on social media in which he explained her death to exceeding pressures of her professional life as a junior physician in the Norwegian public healthcare system. This personal story fueled a nationwide movement among Norwegian physicians who under the hashtag #legermåleve (#doctorsmustlive), took to social media and news outlets and collectively warned against what they considered unsustainable working conditions in the public healthcare system. Together their accounts depicted an unsustainable, unbearable, and irresponsible work environment, converging in a strong call for systemic reform.

### Thinking along

“Thinking along” is the first step in Vosman and Niemeijer’s methodology, where one is to closely engage with the lived experiences and perspectives of those involved in care practices to understand their experiences and challenges from within, without imposing external theoretical judgments.

The stories shared as part of the #doctorsmustlive movement reflect the lived reality of physicians and serve as powerful testimonies to their struggles. A compilation of firsthand accounts (Blindheim and Zerener 2023) reveals the personal and professional toll of their work, highlighting themes such as workload, competency, adequacy, responsibility, and suffering. One recurring theme is the overwhelming workload and constant time pressure faced by physicians. Many describe situations where the demands of their roles leave no time for basic needs. As one physician stated: “I often don’t even have time to go to the toilet, eat, or drink”. Closely tied to this is theme of competency, or rather lack there of, and fear of failing patients due to insufficient support, training, and preparation. Physicians recount a lack of supervision and resources, leaving them feeling unsupported in critical moments. One physician shared: “I felt like I wasn’t measuring up and was afraid for patient safety”. Another described an isolating and harrowing experience: “I came in for the night shift and was told there was no one to cover. When a critically ill patient arrived, and I needed to consult, the supervising doctor didn’t answer the phone. I broke down crying, feeling completely alone and terrified for the safety of the patient”. A third prominent theme was inadequacy, as physicians expressed pressure to be constantly available, even at the expense of their own and their families’ well-being. Taking time off was fraught with guilt and consequences, as one physician explained: “It wasn’t easy to call in sick. It would affect my colleagues and wasn’t popular. If I got sick, I would dread calling in for hours, or even the whole night, knowing it would cause problems for others on shift”. The systemic demands placed on them were summed up by another physician: “To be considered ‘suitable’ to become a pediatrician, you should ideally put both your own and your family’s health aside to ensure the hospital’s operations and prioritize a high number of patient consultations over good and safe care. This makes me discouraged and a bit sad. These are not the kind of pediatricians I want my own children to encounter”. Underlying all these themes is a profound sense of responsibility– the physicians communicated that they deeply care for their patients but feel unable to respond adequately due to systemic constraints. As one testified: “I have sustained chronic injury because of this system.” Finally, the strongest theme that emerged was one of suffering. Physicians spoke of feelings of despair and a longing to escape. Many described the emotional toll of their work and the desire to find relief, no matter how extreme. One physician testified: “Had I known this beforehand, I think I would have chosen a different profession. Not because I don’t like medicine, but because the working conditions feel tough, and the sense of inadequacy toward the patients is painful.” Another revealed a longing for an even more extreme escape: “I often hoped to be hit by a car, get sick, or break an arm to avoid going to work. If I had to go on vacation, I secretly hoped the plane would crash. Giving up wasn’t an option” (Blindheim and Zerener 2023; Blindheim et al. 2023).

In the articles and social media posts constitutive of the #doctorsmustlive movement the workload was characterized as not only too large but also to a high degree consisting of inappropriate tasks, that is, tasks that only serves bureaucratic purposes and do not contribute to health. In their view, time constraints, work pressures and institutional rigidity together cause a wide range of negative outcomes in patient care, in healthcare delivery systems and even in the health of the healthcare providers themselves. Their concerns included fear of more medical errors, failure to meet regulatory demands, compromised treatment outcomes, reduced efficiency, moral injury, fatigue, and poor health outcomes for healthcare professionals themselves as the work environment injured their mental and physical integrity.

Collectively they referred to a ‘sick system’, with ever increasing amounts of work and business at the core of the problem. The main remedies they proposed to this sick system was to reduce workload and alleviate and mitigate the time constraints on doctors, for example by increasing the number of doctors and by reducing the number and size of inappropriate tasks or delegating them to other personnel (Espegren 2023).

### Counter-think

The second step in rethinking care is to “counter think,” that is, to stay with the concerns raised but propose other analytical perspectives on them to understand the issues better.

Staying with the physicians’ concerns, one may take a step back to make the phenomenological observation that their essential problem appears to be one of suffering. They report suffering at work (and in their lives), describing their career choice as unbearable and unsustainable. In their own words, this suffering is attributed to the workload. By moving from suffering as an observation to using it as a heuristic, one can begin to question the assumption that the primary cause is merely the quantity of the workload. This includes questioning the perceived burden itself, how the workload of physicians compares with other professions, and how it has evolved over time, as well as exploring the nature of the workload itself and the context in which it occurs. Finally, it involves exploring how the workload may be experienced as perceived threats to personal and professional identities influencing their ability to endure its weight. This approach makes it possible to explore how existential, relational, systemic, moral, and epistemic dimensions of the healthcare environment contribute to physicians’ distress, moving beyond a purely quantitative explanation of their burden.

To critically examine the concerns raised by the #doctorsmustlive movement, it is essential to question underlying assumptions, including how physicians’ workloads compare both across professions and over time. While few studies compare the workload of physicians with other professions, claims of an unbearable workload among doctors can be contextualized by considering various measures of workload across different professions. These measures may include not only weekly working hours, shift duration, and rest periods between shifts, but also levels of responsibility, emotional and physical strain, work-pace variability, environmental factors, autonomy, and task complexity. While many healthcare providers experience many of these factors, the extent may vary significantly between specialties, and observations suggest that some professions may experience comparable or even greater workloads than physicians, depending on how these factors are measured and evaluated (NOU 2016: 1, 2016).

Longitudinal evaluations of Norwegian doctors’ working conditions from 1994 to 2012 indicate that working hours remained relatively stable, consistently staying below the limits set by the European Working Time Directive (EWTD) (Rosta and Aasland 2014). However, between 2016 and 2019, there was an increase in the proportion of doctors working more than 48 h per week, especially among male and female general practitioners and hospital doctors in leadership roles (Rosta and Rø 2023). Despite healthcare reforms and increased work demands, overall job satisfaction remained relatively high (Nylenna et al. 2005; Aasland et al. 2010) and stress levels relatively stable, though there has been a slight decline in satisfaction and an increase in stress over the last decade, with some groups experiencing these changes more acutely than others (Rosta et al. 2019, 2020). There is a scarcity of studies comparing medical professionals across countries. With regards to such comparisons the discussion has focused not on excessive work but rather on whether Norway’s highly regulated conditions - where the standard working week is limited to 40 hours and hospital doctors typically work 45–48 hours– meet international standards for quality practice and skill development, which is often argued to require longer hours in other countries (Rosta and Aasland 2014). However, in some studies the well-being of doctors has been investigated in relation to workload. In a series of comparisons between German and Norwegian doctors, the German physicians reported longer work hours and poorer health. Despite facing professional pressures, Norwegian doctors reported higher overall job satisfaction, which were attributed to better working conditions, supportive organisational structures, and health policies that emphasize work-life balance (Rosta and Aasland 2011; Voltmer et al. 2024). To some extent, then, there is a discrepancy between the presented research on physicians’ health and their vocalization of increasing dissatisfaction with the medical profession.

Another approach to *counter-thinking* the concerns of the #doctorsmustlive movement is to critically examine both the framing of the issue and the proposed solutions. Taking a step back, it becomes evident that the framing of the problem and many of the solutions proposed by the #doctorsmustlive movement are unlikely to gain neither political nor widespread societal support in Norway. Norwegian physician density doubled from 1991 to 2021 and is among the highest in the world (Saunes et al. 2020). The density of the combined skilled health workforce now exceeds 2.5% of the entire population. Public health expenditure has continued to increase both in absolute numbers and relative to Gross Domestic Product (GDP), and recent data on Domestic General Government Health Expenditure Per Capita shows that the Norwegian value exceeds 6,000 USD, only surpassed by the USA. At the same time, with advances in medical science, the number and cost of available medical procedures and treatments continue to increase, leading to a higher health gap: A growing disparity between what is achievable or available in terms of medical procedures and treatments and the actual outcomes or access for individuals within the healthcare system. Accordingly, Norwegian health policies resemble those of the European Union in that they aim for increased efficiency in the future and a stop or at least deceleration in the growth in health workforce density. From the political perspective, the health sector cannot continue to expand without some limits to growth (NOU 2023: 4, 2023). The proposed political solution of simply multiplying the number of doctors and reducing their individual workload therefor runs counter to the dominant policy discourse and also to its diagnostics, as arguably the physicians’ work environment compares well with that of other professions in these terms (NOU 2023: 4, 2023).

Still, questioning the assumptions of increased workload and its supposed incomparability, or highlighting the limitations of the quantitative framing with regards to its relevance and effectiveness, cannot change the matter of fact that large numbers of especially younger Norwegian physicians– as witnessed by the size of the mentioned Facebook group– appear to experience their work situation as unbearable. In this act of *counter-thinking*, however, one may suggest that the framing of their concerns in terms of the quantity of the workload and the magnitude of the work pace is inadequate. The claims are in need of a different framing.

Staying with the heuristic of suffering, this need invites a focus on the qualities rather than the quantities of the burden, exploring how the workload itself may have evolved and how it relates to or even threaten the integrity of physicians and their professional roles and goals. This approach is supported by the fact that the qualities of medical work itself are undergoing significant transformation, continuously reshaped by evolving practices, interdisciplinary collaborations, and rapidly shifting socio-material realities. In this process, physicians themselves are adapting, continuously renegotiating their traditional narrative identities within the fluid and fragmented conditions of what the sociologist Zygmunt Bauman has termed liquid modernity (Bauman 2000; Bowker-Howell et al. 2024). Another approach to *counter-thinking* the concerns expressed in the #doctorsmustlive movement is therefor to trace them to modernisation of medicine itself. Shaped by countervailing forces– the state, the market, and patients– and through standardization and increased bureaucratic control, modern medicine is characterized by a more collective and regulated approach at the expense of the traditional roles and autonomy of the medical profession (Aasland 2015). New generations of physicians may end up in a position between these forces and in tension between generations and rapidly changing expectations and commitments (Bowker-Howell et al. 2024).

Late modernity, characterized by increasing specialization, bureaucratization, and the acceleration of social and professional demands (Beck 1992; Rosa 2013), has restructured the nature of work across sectors, including healthcare. Physicians, once seen as autonomous professionals with control over their practice, now find themselves navigating highly regulated, fragmented systems that prioritize efficiency and measurable outcomes over relational and moral dimensions of care. Time spent on direct patient care have significantly decreased over the last two decades (Rosta and Rø 2023; Rosta and Aasland 2016). Currently most of the GPs’ working time is spent on patient-related activities, but only about half is spent on face-to-face consultations (Morken et al. 2019). Thomassen et al. (2017) analysed this phenomenon (among nurses) as a matter of violation of their professional integrity. Drawing on Sennett’s concept of “craftwork”, they argued that the results of neoliberal reorganisation of care organisations was that the nurses no longer could do their work in the way that they had been taught and by the standards that they considered appropriate and responsible (Thomassen et al. 2017). This analysis may also hold merit when applied to the evolving practices of physicians, suggesting that they, too, face challenges in performing their work in ways aligned with the standards and values they were trained to uphold. Deep-rooted cultural imaginaries are still strong in popular culture (think of the popularity of the TV series Grey’s Anatomy) and, judging by my own time in medical school, still strong in medical education. These imaginaries portray doctors as the supreme caretakers and problem-solvers for patients in need, as empowered leaders who possess the knowledge and capacity to take responsibility not only for the patient at hand but for the patients as collective. A “good doctor,” as viewed by Norwegian hospital doctors, embodies this professional dedication, characterized by a strong presence at the workplace and a robust capacity to work intensively and effectively, often going beyond regular working hours (Hertzberg et al. 2016). Reality, however, is different, not just because healthcare demands continue to outgrow supply but also because better medical science and technology leads to ever more refined expertise and accordingly, increased differentiation and specialization within the medical enterprise. There is no longer one doctor who is omniscient and omnipotent. Instead, patients move on through the complex system of the modern hospital and other care organisations, and their supreme caretaker– to the extent that the entity exists– is an ad hoc multidisciplinary team with ever new members, or even just an algorithm deduced from a set of clinical guidelines. By the success of the modernization processes of scientific research, technological development, rationalization and differentiation, the role of the physician has been reduced from a demigod to something resembling a line assembly worker. This was not what the medical student was prepared for (Aasland 2015). Hence, the work experience is not only one of surprise or even shock (Johansson et al. 2001) but may even amount to alienation, disempowerment and helplessness, and of feeling being dehumanized in a worklife for which one had high, idealistic expectations. This, in turn, can result in a deeper loss of meaning, identity, and a sense of a fractured professional integrity (Cole and Carlin 2009).

For physicians, this is not only a sociological analysis. This restructuring is part of a lived reality - one that is experienced, endured, and felt - one in which the physicians themselves are reshaped (Bowker-Howell et al. 2024). Late modern conditions change what physicians do, the practices they engage in, and the relational spaces they move through, altering what they are woven into as part of the healthcare fabric. Traditionally, physicians have been central as knowledge bearers, doers of good, and stewards of responsibility– not only for individual patients but also for the moral and epistemic dimensions of care itself. Together, knowing, doing good, and being responsible are deeply intertwined with the narrative professional identities of physicians (Cassell 2004). Under the conditions of late modernity, however, all these dimensions are increasingly threatened– arguably threatening the integrity of the physicians themselves.

Late modernity, with its focus on specialization, bureaucratization, and measurable outcomes, has not only restructured the nature of work in healthcare but also shifted and narrowed the ways in which knowledge is created and accessed, impacting physicians as knowers by constraining their epistemic scope and privileging formalized, standardized knowledge over tacit expertise, this in turn influencing physicians’ experience of competency, legitimacy, and adequacy. First, in highly specialized healthcare systems, physicians frequently confront questions outside their areas of expertise, and patients are often referred to someone “better suited”– a specialist with deeper knowledge in a particular domain or a more experienced colleague, leaving physicians and their patients with the sense that “it would have been better for you to have seen someone else”. Rather than fostering an experience of being “good enough” in their roles, this dynamic fragments the physician’s sense of competence and relational authority. Second, positivism formalized into institutional frameworks such as evidence-based medicine, clinical guidelines and advanced technologies constrain medical knowledge into quantifiable and standardized algorithms. This emphasis on measurable outcomes aligns with what (Foucault 1973) famously termed the “clinical gaze”, which prioritizes objective, detached observation over relational and contextual understanding (Foucault 1973). This shift increasingly locks physicians into this gaze, compelling them to view patients in a reductive manner that abstracts individuals into cases, data points, or symptoms. As a result, physicians become not only detached from their patients’ lived experiences but also alienated from their own subjective and embodied experiences as caregivers. Together these frameworks reduce the physician’s role to one of compliance and execution, reducing opportunities for empathic, critical and creative problem-solving. Third, the very nature of what knowledge counts in medical practice is shifting. Physicians are no longer primarily tasked with knowing what to do in a given situation but increasingly with predicting what will happen. This shift from definitive action to probabilistic forecasting introduces new forms of uncertainty, reflecting limits of predictive models. Lastly, the erosion of relational care practices, such as face-to-face consultations and continuity of care, has curtailed the tacit, experiential knowledge that arises from the provider-recipient relationship. The dynamic interplay of empathy, attentiveness, and lived realities has historically been a cornerstone of medical practice. It once informed the physician’s understanding of the patient’s needs while reinforcing their own sense of purpose and professional identity. However, in late modernity time pressures, bureaucratic demands, and task fragmentation encroach on these interactions, undermining the conditions for such knowing. In sum, the suffering of physicians, therefore, can also be understood as epistemic in nature. The constraints imposed by specialization, standardized frameworks, predictive models, and diminished relational care not only challenge physicians’ ability to act competently and confidently but arguably also undermine their sense of professional identity and purpose threatening the integrity of the physician as a knower.

The erosion of the physician as a knower reflects a broader decentering of the physician-patient dyad within healthcare systems. This decentering is not merely a shift in individual practice but part of a larger transformation in how healthcare is conceptualised, structured, and delivered. Central to this transformation is the expansion of medicine’s scope, integrating health not just as a concern of the physician-patient dyad but as a political and societal objective aimed at groups and populations. This integration has blurred the boundaries between clinical care and public health, reshaping not only what is considered “good” in medical practice but also fundamentally altering how physicians experience themselves as moral actors and doers of good. In the Norwegian context these shifts are deeply tied to the utilitarian ethos embedded in welfare states and reinforced by the principles of New Public Management (NPM). Norwegian physicians seem to implicitly adopt a medicalized utilitarian position where the main aim, if not the raison d’être itself, of the healthcare system is to maximize health in the population by some metric, be it QALYs or others. A Foucauldian might say that they display the governmentality typical in the biopolitical state, where physicians adopt and implement the state’s priorities, focusing on controlling and improving the health of populations through policies and systems rather than prioritizing individual care (Suijker 2023). This seems to be a powerful explanation for why these young Norwegian physicians cannot carry the burden of their work: The results will never be satisfactory. By working harder, by working more, one might always help one more patient and producing some more health. One doctor related that she suffers from kidney failure caused by her work: She could not take the time during workdays to drink some water, because that would be to fail the patients (Blindheim et al. 2023). By perverse implication, to go home from work, or to spend some extra time with one patient, is to injure or kill somebody else by not helping them. The experience is one of not being able to help enough patients and help them well enough within the time allotted to work, and this is what creates moral stress and injury and subsequently health problems. The sense of not being a doer of good “enough”, coupled with the threat to moral integrity, may be even more pronounced in affluent welfare states for several reasons. First, the panorama of illness and disease has shifted. It is less a matter of “simple” medical problems for which there might be clear answers on what to do, and even a cure. It is more a matter of chronic ailments, life-extending treatments of old patients with incurable diseases, medically unexplained symptoms and complex psychosomatic conditions. Second, the restructuring of the provider-patient relationship increasingly highlights the tension between the individual rights of patients to choose, the moral obligation of the physician to produce health, and the broader concerns of society, where societal goals, such as resource allocation and public health imperatives, may conflict with individual preferences or professional advice (Mol 2008). Third, the erosion of the physician as a doer of good is closely tied to the transformation of clinical spaces and the decentering of the patient-provider dyad. Returning to Lévinas’s philosophy, the clinical space represents a unique site for ethical engagement - a face-to-face encounter where physicians respond to the irreducible humanity of the patient as “the Other” (Levinas 1988). However, as Mol (2008) highlights in *The Logic of Care*, modern healthcare increasingly prioritizes the logic of choice over the logic of care, framing patients as autonomous decision-makers rather than relational beings requiring ongoing, adaptive care. This shift, coupled with systemic pressures such as standardization and efficiency imperatives, reduces the clinical encounter to transactional interactions, further undermining the relational and ethical dimensions of care (Messinger and Das 2023).

In this context, the suffering reported by physicians can be understood as arising from the tension between three competing notions of ethics: the utilitarian imperative to maximize population health, the ideal of individual autonomy and choice, and the tacit, relational experience of care as an embodied practice of doing good.

This tension is further complicated by the move toward proactive and preventive medicine, where care increasingly focuses on engaging with and production of perceived health risks. As a result, medical care is not only productive of health and comfort but also generates risk, uncertainty, and side effects, reflecting broader features of what Ulrich Beck has termed the “risk society” (Beck 1992). In this context, physicians are not only healers but also producers of risk, as their interventions– whether diagnostic, therapeutic, or preventive– carry inherent uncertainties and unintended consequences– both to the individual and society. Reflexive modernity, another theoretical contribution by Beck (1992), helps illuminate what this means for the physicians themselves who’s role no longer is solely about curing or alleviating suffering; it now also involves navigating the unintended consequences of medical interventions and critically assessing their broader societal implications. Compelled to continuously reflect on their practices, balancing the potential benefits of their actions against emerging risks, physicians are increasingly faced with questioning not only the ethical and moral foundations of their decisions but also their very position in society, further embedding physicians within the paradoxes of late modernity. More is done, and more is asked for, but clinical benefits and medical purposiveness is less certain.

Returning to suffering as a heuristic and the recurring tension between suffering understood as passivity and an opening, one may argue that under late modern conditions, the suffering of physicians arguably aligns more closely with the former, a sense of being bound. Again Beck (1992) provides valuable resources to explore this “boundness” through his concept of “organized irresponsibility,” which refers to the systemic diffusion of accountability within complex institutions like modern healthcare systems. Responsibility becomes fragmented across multiple layers, making it nearly impossible to pinpoint accountability for systemic failures. Physicians, as central actors within these systems, often find themselves cought in this web of organized irresponsibility where they are burdened with the outcomes of systemic shortcomings yet lack the resources to address the root causes, compounding their sense of helplessness and suffering.

The #doctorsmustlive movement focused on reducing the quantity of the burden– the load– or sharing it with others; however, comparison with other professions and over time indicates that this causal attribution may not be the key to understand the specificity to the situation. *Counter-thinking* however, drawing on the social theory of late modernity, suggests that rather than merely an issue of excessive workload the burden faced by physicians may be better understood as a symptom of broader societal and institutional transformations that threaten them not only as providers but also as persons, contributing to their suffering. The qualities of the workload not only undermines their sense of professional identity but also contributes to epistemic and moral injury and a pervasive sense of being bound– un-able to respond, as they are unable to reconcile their tacit ways of knowing and ethical commitments with the positivist and utilitarian logic that governs the modern healthcare systems in which they are embedded. This may reflect how the burden of the workload has become unbearable, as the work has become less gratifying, less rewarding, less generative, depriving them of the meaningful experiences of knowing, doing good and being responsible that once sustained their ability to carry the burden of care.

### Rethink

The third step in rethinking care is where the gaze is opened up from the original concern to seeing all who deserve to be cared for in the matter at hand. As Vosman (2018) argues, care ethics should not only respond to immediate relational needs but also critically engage with the institutional frameworks that configure these relationships (Vosman 2018). This perspective invites deeper reflection on the trade-offs embedded within these systems of thought, practice and institutional structure: Is there an inevitable trade-off between caring for patients and caring for physicians and other health personnel? Is it so that the only solution to the growing health gap in late modernity is to find the optimum at the intersection between curves of supply and demand, where the rate of added health benefit for patients equals the rate of health deterioration among the healthcare workers? I do not think so, and neither does the physicians themselves.

Outlines of solutions are inherent in the #doctormustlive movement itself, others in biomedical discourse. For one, the emergence of movements like #doctorsmustlive marks a significant shift in the professional identity of physicians. While senior consultants deeply integrate their professional identity into their lifestyle, younger physicians tend to view their work merely as a job (Hertzberg et al. 2016). A movement of physicians formed around complaints about their own situation and own health, seems unthinkable only a few decades ago, departing significantly from traditional notions of the medical profession as empowered, autonomous professionals who embody self-efficacy, resilience, and a strong sense of purpose. The rise of movements like #doctorsmustlive reflects a parting from these narratives, as physicians increasingly vocalize their vulnerability and frame themselves as sufferers within the same system they serve, adopting narratives and roles traditionally associated with those they treat, positioning themselves as subjects of systemic pressures and medicalized discourses of stress, burnout, and mental health.

At the same time, physicians are being reimagined– and reimagin themselves– as increasingly removed from the strain of care itself. This is reflected in sociotechnical imaginaries of a future medical system in which utilitarianism is brought to its logical endpoint– a medical system where healthcare professionals are highly skilled and specialized technicians in service of big data and automated decision-making processes, a system where medicine is transformed to pure mechanics and engineering (Breuer and Müller 2024; Rowland et al. 2024). Indeed, when the American Society for Clinical Oncologists set out to envision the future of oncology in 2030 they optimistically portrayed a future in which oncologists do not meet patients but rather function as data experts and controllers (Strand 2022). In that case, the professional role of the physician will have changed so much as to have almost nothing in common with our current and traditional understanding. There will be no direct patient care left.

Arguably, these emerging solutions are not the result of careful *rethinking* but are instead products of the very suffering they seek to address - expressions of hope: If only one had fewer patients and more time, the patients would have had their problems solved and the physicians’ work would have fulfilled their own and the patients’ expectations. If only one could be distanced from the unresolvable epistemic and moral dilemmas inherent in practice, the pressures faced might seem more bearable. Staying with care ethics one may suggest that rather than resolving the deeper issues of care, these solutions risk perpetuating a system that alienates providers from their patients and from their own professional identities.

To *rethink* such an extreme frame is not difficult. Considering this expanded view of the workload– how it not only burdens but also threatens the integrity of those bearing its weight - and the solutions that emerge under this pressure, another path of reimagining takes shape, one that reflects on what is lost in such transitions. It takes as its starting point the perhaps stereotype professional identities and self-images among doctors as professionals with a high degree of autonomy, self-efficacy, and sense of empowerment, but who also experience the job as a source of social integration and personal meaning. Taking this seriously, not as a stereotype but as an experienced reality– doctoring experienced not as mere strain but as a privilege and a source of profound reward - a fundamental question opens up: Could the very elements sidelined by a utilitarian, data-driven model - relationship-building, empathy, and the craft of individualized care - hold the key to resolving the tensions and suffering faced by healthcare workers? It may even be argued that physicians’ own medical knowledge would be critical to the seemingly determinist idea that high workload by necessity causes workers to become victims of poor health outcomes (Burgard and Lin 2013). Indeed, as medical practitioners themselves they might frequently argue towards their patients that work is a source of health and that there is no linear causality from workload to poor health. Could we reimagine a return to such practices, or has the system moved so far that exploring this question is a luxury we can no longer afford– lacking the resources, time, and imagination to bring these values back into the heart of healthcare?

The question in early 21st century welfare states is to what extent there can be any restoration and return to the craftwork it was once conceived. In that regard, there might be more grounds for optimism for nurses than for physicians. It seems reasonable to argue that several of the key roles of nurses, such as being patient caregivers, educators and advocates, remain present also in modern hospitals and other care organisations, even if pressed on time and threatened by increasing loads of paperwork (or digital work). For young physicians especially in hospital settings, however, the discrepancy between their imaginaries and expectations and the reality of work life may be daunting.

What is conspicuously lacking in the framing of the issue so far, however, is the critique of medicalization that has been part of critiques of late modernity for almost half a century (Armstrong 1983; Conrad 2007; Illich 1976; Rabinow and Rose 2006). The #doctorsmustlive movement seems to be as unimpressed (or ignorant) of these critiques as are Norwegian (and European) health authorities, in that they uncritically assume that more healthcare (barring medical error due to excessive workload) leads to better health and better lives. Critiques of medicalization have produced abundant arguments why more healthcare does not necessarily imply better health, and why better health cannot be equated with better life and better society. Instead, the expansion of medicine, as a driver of late modern conditions itself, has been charged with perpetuating the very suffering it seeks to alleviate. In this sense, rethinking through the lens of suffering offers pathways for envisioning institutional reforms.

Rethinking the frame in concrete terms implies to make a ground for claiming that physicians are not acting immorally when they take time to eat, drink water and otherwise care for their own life. It also implies to challenge the totalitarian ambitions implicit in the call for continual growth of the healthcare sector. By reducing the load - both in terms of quantity and in reframing the expectations placed on healthcare workers - a space can be reclaimed for care as a relational, ethical, and restorative practice. This would allow physicians to reconnect with the meaningful aspects of their profession, fostering environments where care is not just delivered but also experienced as deeply human and humane.

In intellectual terms the task is easy; however, in practical terms it is difficult, especially in late modern, secularized welfare states where help from society to individuals increasingly is channeled via the healthcare system and where the individuals have to frame their problem as medical ones in order to receive help. The #doctorsmustlive movement is in this way a telling illustration from a society that became so medicalized that medical work became experienced as unbearable by some of its practioners, and where claims of adverse health outcomes were leveraged in order to bring attention to the problem. As such, the #doctorsmustlive movemenent embodies a paradox inherent in the risk society (Beck 1992), where physicians, as both sufferers and responders, are caught in a double bind: their suffering is framed as a health issue to be resolved within the existing healthcare paradigm making them caught up in the very system they both produce and are produced by. Yet, within the #doctorsmustlive movement itself there are openings. A key example is the framing of physician suicides within the movement. Rather than viewing these tragedies as consequences of individual mental illness, a critical perspective resists medical frames, asserting such suffering as deeply tied to systemic and social conditions. As Theodor Adorno suggests, suffering has the potential to critique and expose the contradictions of the systems that produce it (Adorno 1973). Following Lévinas, suffering represents more than a symptom of systemic dysfunction; it serves as an ethical opening - a call that demands engagement. For Lévinas, this engagement requires taking suffering seriously, not as a problem to be solved but as a relational phenomenon that demands a response. The suffering implicit in the #doctorsmustlive movement thus functions as both a powerful critique of late modern healthcare and its priorities and as a potential opening for rethinking and reimagining care.

## Discussion: reclaiming the place for care

In this paper I have engaged in the philosophical venture of *rethinking care.* It should be noted, however, that this was not a given. To infer directly from the presence of care in the phenomenon to the need for care ethics or care theory, would be an equivocation and conflation of levels. It would have been and still is entirely possible to engage with the phenomena at face value or to theorize and rethink care practices in care organisations without care ethics or care theory. The concerns brought forward in the #doctorsmustlive movement intersect with broader labour rights issues and could be understood at face value as such, reflecting not merely testimonies to suffering but also being inherently political, narrated in ways that aim to mobilize change. Another alternative would be to use other theoretical resources. Thomassen et al. (2017) provided a sociological analysis of Nordic work life as threats to professional integrity, by means of Sennett’s concept of craftswork (Thomassen et al. 2017). Others have suggested the negative effects of neoliberal ideology on organisational culture in healthcare organisations (LaGuardia and Oelke 2021). From Bordieuan field analysis (Bourdieu 1993) to Foucauldian discourse analysis (Foucault 1972) and beyond, there are ample resources in sociology and social theory that could have been fruitfully employed on a case such as the #doctorsmustlive movement. As much as systematic studies of, say, biopolitics and governmentality in and around contemporary Norwegian healthcare institutions would be timely in light of #doctorsmustlive, the point of departure for this paper has nevertheless been developments in care ethics and care theory. Care is not only the subject, theoretical framework, and methodological resource of this paper but also its underlying assumption and premise– a lens through which clinical spaces are understood as deeply human and humane. Thinking from, through, and with care brings into focus how, in late modernity, more is at stake and in motion than mere materiality and structure; care encompasses knowledge and thought, relational dynamics, ethical obligations, and the shaping of both professional and personal identity.

Building on this premise, this analysis draws on and connects to several ongoing conversations in the anthropology, sociology, and philosophy of medicine, acknowledging that each perspective warrants deeper exploration– endeavors that extend beyond the scope of this text. Instead, the goal here is to sketch a broader landscape, outlining connections and tensions that invite further elaboration. By engaging with these diverse traditions, the analysis contributed towards mapping the intersections where social theory and care theory converge, offering a framework to explore their mutual influence and challenges.

As such, this analysis is not adept nor attempts to explain the breadth and depths of healthcare providers’ unsettling experiences or concerns, and many dimensions of healthcare personnel’s experiences of discomfort, inadequacy, and pain are not brought to the forefront in this paper. Rather, the purpose has been to invite a deeper reflection on how temporal aspects of caregiving interact with epistemology, ethical thought and experiences, potentially reshaping understandings of what constitutes good care in a late modern context.

By foregrounding these expressions of concern of physicians I have also tried to foreground– through the operations of *counter-thinking* and *rethinking*– some pressures exerted upon them in the larger developmental processes of restructuring healthcare. These processes entail a shift from a relational practice comprised of provider-recipient dyads, where the physician is allowed to give care in meaningful ways, to a social ordering of medicine as a social practice, decentering, not only of patients but also of the professional actors. Norway and the Scandinavian welfare states are interesting in this sense as perhaps the prime examples of late modern social democracies where healthcare is not only profoundly modern in the scientific and technological sense but also is explicitly tied to a utilitarian understanding of equality. To somewhat simplify, equality in Scandinavian social democracy is conceptualised as the ideal of *equality in outcome*, not just in terms of equal opportunities. For example, in prioritization of Norwegian health services, severity is an independent criterion introduced to counteract the unfairness of grave disease (Shah 2009). This ideal arguably poses unattainable demands on the healthcare system, where enough is never enough, or rather, services are never good enough if anyone lags behind in the health outcomes. Accordingly, healthcare workers are not only supposed to care for their patients. They are also expected to serve as tools for an idealized utilitarian biopolitical system– they should be equalizers. The story about the kidney failure could be taken to be a reductio ad absurdum of its excessively utilitarian and actually totalitarian character where there is no room for other values, not even personal self-care. In the Hippocratic oath, the physician swears that “into whatsoever houses I enter, I will enter to help the sick”. Nowhere in the oath is it promised to enter all houses, or enter houses without cessation. Something has gone wrong if physicians in routine work take the duty to help the sick (or the utilitarian goal of maximizing patients’ health) to imply that they should always work or that they are not entitled to spend some seconds to drink water during their work. I note again that this was happening in Norway in peace time, in the absence of any other health emergency.

While these reflections emerge from a specific corner of the world and at a specific time, many of the dynamics are, however, more generalizable.Echoing international concerns about how healthcare workers meet work demands that have increasingly intensified over time (West et al. 2018), the presented case study from Norway offers valuable insights into the pressures faced by healthcare systems and workers in late modern societies globally. Solutions traditionally offered to address these pressures often focus on supporting the individual practitioner though efforts such as mindfulness training for individual resilience and organisational support to improve workplace conditions (Sipos et al. 2024; West et al. 2018). In the context of this analysis, such solutions could be interpreted as symptomatic of medical practitioners themselves being subject to medicalization - framing the problem as residing within the individual rather than in the world they inhabit. These approaches risk stabilising and solidifying the *status quo*. By focusing on individual adaptation rather than systemic transformation, the solutions themselves are manifestations of “organised irresponsibility,” diffusing accountability and leaving the deeper structural and relational issues unaddressed.

By taking a philosophical and critical approach, this analysis opens up possibilities for rethinking care at a deeper level. By challenging prevailing assumptions and reframing care imaginaries, this paper calls for a broader interrogation of the systemic, relational, epistemic and moral dimensions of healthcare work in late modernity.

As such, the #doctorsmustlive movement can be interpreted as an expression of the suffering that emerges as physicians’ possibilities to act as caregivers are being eroded. One could argue that they are vocalizing and reacting to experiences of being decentered, disempowered, reduced as *carers*, as knowers, as moral actors, as responders. Through this lens, physician suffering highlights not just failures within the healthcare system but also the broader societal logic that prioritizes growth, efficiency, and measurable outcomes over relational and moral dimensions of care. What results, I have argued, is a confrontation between certain visions, imaginaries, desires, and the subjectivities they shape with the very realities from which they arose. And this confrontation is itself one of suffering. It is exactly at this point the place for care, and for care ethics, should be reclaimed. Staying with Adorno and Lévinas engaging deeply with these visions, imaginaries, desires, and subjectivities– as well as the suffering they both produce and reflect– offers an opening, a critical pathway to understanding and addressing inherent challenges of late modernity itself.

From the perspective of care ethics, the challenge is not solved by simply enlightening the suffering physicians so that they understand that their profession is changing and telling them that the longing to care for their patients is a case of romantic nostalgia. Neither is the challenge solved by embracing the Utopian utilitarian regime in which human caregivers are envisioned replaced by algorithms, technology and ultimately welfare robots. With the care ethics and care theory traditions I would argue that this would be a gross misunderstanding not only of what a good doctor is, but what a good life and fundamentally what a human is. We should accordingly resist reductionist biomedical imaginaries that claim that algorithms and robots are epistemically and morally sufficient (Engen 2022). Rather than accepting the inevitability of becoming or being substituted by robots, the care perspective would insist on the need to reclaim the place for care, acknowledging that the wish and the need to care for others is humanity at its best and a virtue to be embraced and cultivated. This is not an innocent claim. As Puig de la Bellacasa has explained, care is never innocent as caring for something means caring less for something else (De La Bellacasa 2017). It entails opposition against the utilitarian project of prioritizing equality in health outcome, at least as a goal to be pursued at the level of the individual healthcare worker. This may seem abstract and political but is also a matter of concrete advice. If we return to the example of the physician who could not find time to drink water and accordingly developed kidney failure, we could advise her colleagues to claim the place and take their time for care. This would include time to properly care for the patient in front of them but also time to proper self-care.

This also requires reimagining the concept of responsibility in healthcare and society. Moving beyond rigid notions of responsibility– anchored in accountability through legal and institutional frameworks or universal ethical norms - we must embrace the concept of response-ability, as articulated by Donna Haraway (Haraway 2016). Response-ability involves cultivating the capacity to respond to calls for care: for patients, for colleagues, and for oneself. It is not merely the ability to act but an ethical commitment to engage critically with the structures and values that shape care. Haraway emphasizes attentiveness, reflection, and relationality, recognizing the interconnected nature of caregiving and the need to “stay with the trouble” of complex and often troubling realities. This perspective invites a deeper engagement with the ethical and relational dimensions of care in healthcare systems.

This interpretation calls for creating spaces where physicians can explore their experiences of suffering, examine its systemic roots, and uncover its deeper implications. By doing so, they can resist reductive framings of their roles by challenging the utilitarian logic that equates health with well-being and efficiency with effectiveness, advocating instead for a healthcare system that values relational integrity, professional autonomy, and the moral dimensions of care. This shift would not only address the immediate concerns of physicians but also spark a deeper critique of late modernity, contributing towards a broader reimagining of what it means to care in late modern societies. In this way fewer patients will be seen to in the course of a workday. That problem, however, is one that the late modern society should deal with at a higher level of organisation, such as the political level, instead of– to paraphrase Ulrich Beck– dump the responsibility on the individual physician (Beck 1992).

## Data Availability

Data sharing is not applicable as no datasets were generated or analyzed.
